# Identification of two novel variants of the *BCL11B* gene in two Chinese pedigrees associated with neurodevelopmental disorders

**DOI:** 10.3389/fnmol.2022.927357

**Published:** 2022-09-13

**Authors:** Fengyu Che, Xiaoling Tie, Hong Lei, Xi Zhang, Mingyue Duan, Liyu Zhang, Ying Yang

**Affiliations:** ^1^Shaanxi Institute for Pediatric Diseases, Xi’an Children’s Hospital, Xi’an, China; ^2^Department of Rehabilitation, Xi’an Children’s Hospital, Xi’an, China

**Keywords:** *BCL11B* gene, neurodevelopmental disorders (NDD), intellectual disorder, minigene, immunodeficiency

## Abstract

**Objective:**

According to a recent report, the mutation of transcription factor gene *BCL11B* is associated with the development of neurodevelopmental disorders and immune deficiency. By analyzing both clinical features and genetic variations, this study aims to reveal the genetic etiology of four patients with neurodevelopmental disorders from two unrelated Chinese pedigrees.

**Methods:**

From the 4 cases, the clinical data were collected. The potential pathogenic gene variations were analyzed by means of based-trio whole exome sequencing (Trio-WES) and then validated through Sanger sequencing in their respective pedigrees. Furthermore, both the *in vitro* minigene assay and the NMD assay were performed to evaluate the impact of splicing and frameshift variants.

**Results:**

The 4 patients displayed mild-to-severe intellectual developmental disorder, which was accompanied by speech delay, dysmorphic facies, and serious caries. In addition, the extended phenotype of developmental regression was observed in the proband from Family 1, which has been unreported previously. Molecular analysis was conducted to identify two novel heterozygous variants in the *BCL11B* gene: a maternal splicing variant c.427 + 1G > A in Family 1 and a *de novo* frameshift variant c.2461_2462insGAGCCACACCGGCG (p.Glu821Glyfs*28) in Family 2. As revealed by the *in vitro* minigene assay, the c.427 + 1G > A variant activated a new cryptic splice site. As confirmed by an overexpression assay, there was no significant difference in the level of mRNA and protein expression between the mutate-BCL11B (p.Glu821Glyfs*28) and the wild type. It confirms that p.Glu821Glyfs*28 variant could be an NMD escaping variant.

**Conclusion:**

The extended phenotype of *BCL11B*-related disorders is reported in this study to reveal the clinical and genetic heterogeneity of the disease. The study starts by identifying a splicing variant and a novel frameshift variant of the *BCL11B* gene, thus confirming its aberrant translation. The findings of this study expand the mutation spectrum of the genetic *BCL11B* gene, which not only improves the understanding of the associated neurodevelopmental disorders from a clinical perspective but also provides guidance on diagnosis and genetic counseling for patients.

## Introduction

Neurodevelopmental disorders, including intellectual disability and language disorder, have become a major public health concern that places a heavy burden on both families and the whole society (Maia et al., 2021). In recent years, the rapid development of genome sequencing technology has provided an effective solution to diagnosing neurodevelopmental disorders, which is conducive to the prevention of birth defects. *BCL11B*-related disorder has been a recently reported hereditary disease caused by the *BCL11B* gene *de novo* variant. Up to now, there have been plenty of reports on the role of the *BCL11B* gene in the nervous and immune systems (Arlotta et al., 2005; Li L. et al., 2010; Li P. et al., 2010; Kominami, 2012; Yu et al., 2015; Simon et al., 2016, 2020; Fang et al., 2018; Daher et al., 2020), i.e., early studies demonstrate that Bcl11b plays a critical role in the development of corticospinal motor neurons axonal projections to the spinal cord *in vivo* by loss-of-function experiments in null mutant mice for Bcl11b (Arlotta et al., 2005); on the other hand, Bcl11b was expressed in all T cell compartments and was indispensable for T lineage development and commitment in mice (Li L. et al., 2010; Li P. et al., 2010). Those patients carrying the pathogenic variations of the *BCL11B* gene tend to have immune deficiency or intellectual developmental disorder, the major clinical manifestions are speech delay, dysmorphic facies, and T-Cell abnormalities. Meanwhile, it is accompanied by various phenotypes, such as autistic symptoms, attention deficit-hyperactivity disorder, anxiety, dental anomalies, and other behavioral abnormalities (Punwani et al., 2016; Lessel et al., 2018). So far, there have been merely19 cases reported worldwide (Punwani et al., 2016; Lessel et al., 2018; Qiao et al., 2019; Prasad et al., 2020; Yan et al., 2020; Yang et al., 2020).

Located at chromosome 14q32.1, the human *BCL11B* gene encodes a C2H2-type zinc finger protein. The isoform 1 (NM_138576.2) of *BCL11B* consists of 894 amino acids and 4 exons (Lennon et al., 2016). All of these six zinc finger domains are located in the last exon (exon 4), in which the ZnF2 and ZnF3 domains are responsible for DNA binding. Besides, the N-terminal CCHC zinc finger motif plays a vital role in the formation of the BCL11B dimer (Kominami, 2012; Grabarczyk et al., 2018). Currently, there are 17 variants of the *BCL11B* gene identified in 19 patients through different studies. The major types of *BCL11B* genetic variation include frameshift, non-sense, missense, and complex chromosome rearrangement. However, there is still no report on splicing variation.

In this study, 4 patients with neurodevelopmental disorders and extended phenotypes were reported. Notably, a novel splicing variant and a *de novo* frameshift variant were detected through whole exome sequencing from the two studied families separately. Furthermore, the function of the two gene variants was verified by an *in vitro* minigene assay and an NMD assay.

## Materials and methods

### Patients and ethical approval

**FIGURE 1:**
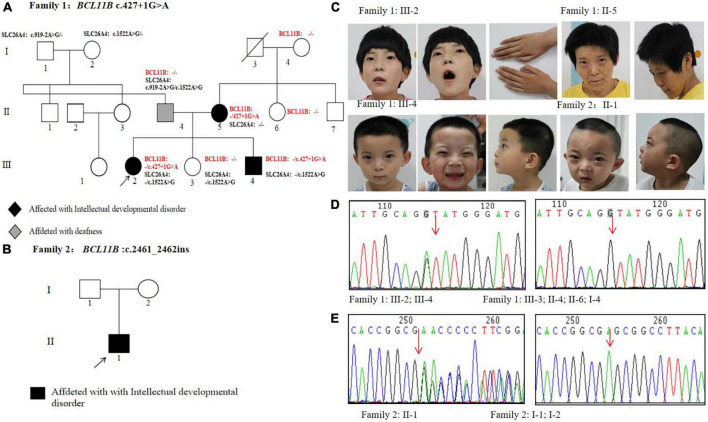
Pedigrees and genetic analysis of two families. **(A)** The pedigree of Family 1 carrying a splice variant c.427 + 1G > A in *BCL11B*. **(B)** The pedigree of Family 2 carrying a frameshift variant c.2461_2462insGAGCCACACCGGCG in *BCL11B*. **(C)** Images of patients with *BCL11B* variants. **(D)** The sanger sequencing of the c.427 + 1G > A variant in Family 1 members. **(E)** The sanger sequencing of the c.2461_2462insGAGCCACACCGGCG variant in Family 2 members.

Two unrelated non-consanguineous Chinese pedigrees involving 4 patients diagnosed with neurodevelopmental disorders were collected. The neurodevelopmental disorders of the studied patients were not caused by other clinical factors, e.g., perinatal brain injury, traumatic brain injury, and infections. In Family 1 (Figure 1A), the proband (III-2) and her mother (II-5) showed severe intellectual disability. Her young brother (III-4) displayed mild developmental delay, and her father exhibited hearing impairment and dysarthria. As an exception, her younger sister (III-3) was healthy. In Family 2 (Figure 1B), the proband presents showed the specific signs of language impairment and mental retardation, but his parents were healthy.

For both families, all the data and analyzed peripheral blood samples were collected after receiving the expressed consent from both the guardians and other participated family members. The study was approved by the Ethics Committee of the Xi’an Children’s Hospital and complied with the Declaration of Helsinki declaration.

### Genetic analysis

About 1 μg of genomic DNA was extracted from 200 μl of peripheral blood by using a Qiagen DNA Blood Midi/Mini kit (Qiagen GmbH, Hilden, Germany) and sheared into fragments. Then, they were hybridized by using the Nano WES Human Exome probe sequence capture kit (Berry Genomics Corporation, Beijing, China). Trio whole-exome sequencing (Trio-WES) was performed for both families. A Burrows-Wheeler Aligner tool was used to align the sequencing reads with the human reference genome (hg19/GRCh37). PCR duplicates were removed through the Verita Trekker^®^ Variants Detection System (v1.57; Berry Genomics, Inc). The third-party software Genome Analysis Toolkit (GATK) was employed to carry out variant calling. Then, all variants were annotated and interpreted through multiple databases, including 1,000 Genomes Project, Exome Aggregation Consortium (ExAC), and Genome Aggregation Database (gnomAD). To better interpret the genetic variants, they were classified into five different categories according to the American College of Medical Genetics and Genomics (ACMG) guidelines (Richards et al., 2015). The suspected variants were subsequently validated through Sanger sequencing in the studied families.

### Minigene splicing assay

**FIGURE 2:**
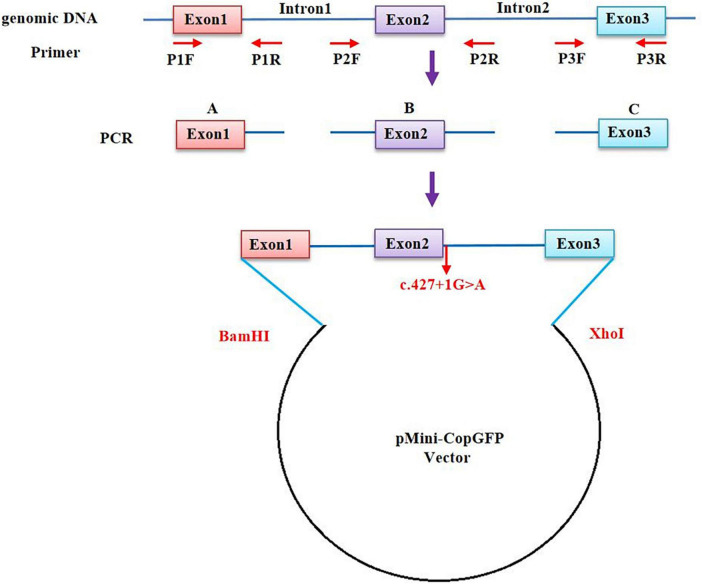
A schematic diagram of primer design for a minigene assay of the *BCL11B* gene. The upstream and the downstream of Intron 1 and Intron 2 were retained, respectively, and the intermediate sequences were removed (NG_027894.1: from g.5780 to g.18155 of Intron 1; from g.20001 to g.44443 of Intron 2).

The *in vitro* minigene assay was carried out to examine the target gene regions covering *BCL11B* exon1-3, intron 1, and intron 2, and they were amplified from gDNA of the proband in Family 1. Due to the large size of the introns 1 and 2 of the *BCL11B* gene, the two introns were split and constructed in plasmid (Figure 2). Three pairs of overlapping primers (P1F/1R, P2F/2R, P3F/3R) were developed to amplify the heterozygous c.427 + 1G > A mutation site from the gDNA fragment by seamless cloning (Vazyme Biotech Co., Ltd., Nanjing, China). Then, the amplified DNA products were recombined and cloned into the two digestion sites (*Xho*I/*Bam*HI) of the pMini-CopGFP vector (Hitrobio Biotechnology Co., Ltd., Beijing, China). Furthermore, the recombinant plasmids pMini-CopGFP-BCL11B-wt (wild type) and pMini-CopGFP-BCL11B-mt (c.427 + 1G > A) were validated through Sanger sequencing (P4F/4R). Subsequently, the plasmids were transfected into HEK293 cells. With total RNA extracted and reverse-transcribed into cDNA after 48 h of transfection, reverse transcription polymerase chain reaction (RT-PCR) was triggered by a primer pair of P5F/5R. Afterward, the cDNA products were examined using 1% agarose gel electrophoresis and further confirmed by means of Sanger sequencing. The length of wild-type cDNA products was determined as 711 bp.

To substantiate the conclusion drawn in this study, the assay was conducted repeatedly in another HepG2cell line by using the same experimental method as above.

### Overexpression of recombinant plasmid carrying frameshift variant (p.Glu821Glyfs*28) of *BCL11B*

The full-length cDNA product of *BCL11B* was synthesized and cloned into the green-fluorescent pEGFP-C1 vector with two digestion sites (*Xho*I/*Bam*HI) so as to obtain a recombinant wild-type plasmid (pEGFP-C1-BCL11B-wt). Meanwhile, a pair of site-directed mutagenesis primers (P6F/6R) was developed to construct mutant plasmid pEGFP-C1-BCL11B-mt (c.2461_2462insGAGCCACACCGGCG) from pEGFP-C1-BCL11B-wt. Both wild-type and mutated plasmids were confirmed by bidirectional sequencing. As per the instruction of the manufacturer, the plasmids of pEGFP-C1-BCL11B-wt/mt (the wild-type group and the mutant group) and pEGFP-C1 (the control group) were separately transfected into HEK293T cells with the assistance of Lipofectamine™ 2000 (Invitrogen, Thermo Fisher Scientific, United States). Each transfection group was replicated in 3 cell wells. After 48 h, total RNA was extracted from the transfected cells by using TRizol Reagent and then reverse-transcribed into cDNAs. Meanwhile, β-actin was treated as the housekeeping gene for processing in the same protocol. The primers for RT-PCR include P7F/7R and human-b-actin-F/R. The cell lysates in each group were collected to obtain total proteins, while the expression of targeted protein was analyzed through Western blotting, which was performed using Bcl-11B (D6F1) XP^®^ Rabbit mAb (#12120, Cell Signaling Technology, United States). All the primers are detailed in Supplementary Table 1.

## Results

### Clinical features

The clinical features of 4 patients were summarized, as shown in Table 1. The phenotypes of the studied patients were consistent with the reported cases, showing psychomotor retardation, the intellectual disability with speech and language impairment, but no obvious immune deficiency. Notably, the proband of Family 1 was able to sing nursery rhymes, recite ancient poems, and make simple communication before the age of 3. However, a significant regression in language and cognitive development manifested itself after Year 3. She also showed the signs of autism and the inability to speak and the lack of common attention and eye contact. In addition, she tended to feel anxious and frightened.

**TABLE 1:** Clinical features of the 4 patients with *BCL11B* variants.

Clinical features	Family 1			Family 2
**General**	III-2 (Proband)	III-4 (Young brother)	II-5 (Mother)	II-1 (Proband)
Perinatal conditions	Normal	Premature infant	Normal	Neonatal jaundice
Age at diagnosis	11 y	3 y 10 m	35 y	3 y 8 m
Gender	Female	Male	Female	Male
Degree of ID/DD	Severe	Mild	Severe	Moderate
Motor dealy	Mild	Mild	Mild	Mild
Walked unsupported	2 y	1 y 6 m	NA	1 y 6 m
Current speech	No words, high pitched voice	Short sentences	Short sentences, hoarse voice	3–4 words
Autistic features	+	–	–	–
Behavioral abnormalities	Stereotypic movements, sometimes aggression	No particular	Timid	Problems with changes
**Neurology**				
Muscle tension	Hypertonia	–	NA	Hypertonia
Serzures/Abnormal EEG	+	–	NA	+
Brain MRI	–	–	NA	–
**Facial abnormalities**				
Thin eyebrows	+	+	+	+
Hypertelorism	+	+	–	+
Long philtrum	+	+	+	+
pointed chin	+	+	+	+
Low-set ears	+	+	+	+
Prominent nose	+	+	–	+
Thin upper lip	+	+	+	+
**Others**				
Dental anomalies	Extensive caries	Extensive caries	–	Extensive caries
Feeding difficulties	–	–	–	+
Immune response	Frequent infections	Frequent infections	–	Allergies
***BCL11B* Variant**	c.427 + 1G > A	c.427 + 1G > A	c.427 + 1G > A	c.2461_2462ins

“+” Yes, “–” No, “NA,” not available; y, year; m, month.

All these 4 patients exhibited the common signs of facial dysmorphism, such as thin eyebrows, long and smooth philtrum, thin upper lips, and micrognathia (Figure 1C). The proband in Family 1 was barely cooperative with IQ and autism tests due to poor recognition and adaptability. The proband in Family 2 scored 38.2 in total developmental quotient (DQ) against the Gesell developmental scale. To be specific, the proband scored 44.9 in adaptability, 46 in gross motor, 37 in fine motor, 30.7 in language, and 32 in personal-social ability. Laboratory investigation showed no abnormality among other patients in terms of blood ammonia, blood lactic acid, serum amino acids and urinary organic acids metabolic analysis, brain magnetic resonance imaging (MRI), hearing and vision examinations, except for the proband’s mother (Family 1: II-5, who objected to any further clinical or laboratory investigations in this study). The electroencephalogram (EEG) results of two probands suggested occasional epileptiform discharges mainly in the bilateral temporal lobes, despite no experience of witnessing clinical seizures.

To evaluate the immunity level of the patients, flow cytometry investigation was conducted to indicate an abnormality in the absolute counts of the lymphocyte subset (Table 2). The ratio of CD4 + /CD8 + was significantly reduced not only in the proband (Family 1: III-2) and her young brother (Family 1: III-4) but also in the proband of Family 2 (Ding et al., 2018). As for the levels of immunoglobulins, IgA, IgG, and IgM, they fell within the normal range for the III-4 of Family 1 and the proband of Family 2. By contrast, there was a slight increase in the levels of IgM and IgA for the proband of Family 1, whose IgG level was normal (Supplementary Table 2).

**TABLE 2:** T. B. NK lymphocyte immunophenotype of the patients of this study.

Cells	Family 1: III-2 (Proband)		Family 1: III-4 (young brother)		Family 2: Proband	
	Result	Reference range*	Result	Reference range*	Result	Reference range*
CD45 + (cells/μL)	**4,340**	2020–3,500	4,132	2,790–6,350	4,916	2,790∼6,350
CD3 + /CD45 + (%)	**86**	62–77	**81**	54–73	**74**	54∼73
CD3 + (cells/μL)	**3,720**	1,297–2,480	3,341	1,794–4,247	3,659	1,794∼4,247
CD3 + CD8 + /CD45 + (%)	**56**	23–32	**48**	19–33	30	19–33
CD3 + CD8 + (cells/μL)	**2,424**	509–1,050	**1,995**	580–1,735	1,483	580∼1,735
CD3 + CD4 + /CD45 + (%)	**23**	28–41	**23**	24–43	25	24–43
CD3 + CD4 + (cells/μL)	1,002	621–1,258	945	902–2,253	1,224	902–2,253
CD16 + 56 + /CD45 + (%)	**5**	8–23	8	7–21	10	7–21
CD16 + 56 + (cells/μL)	238	203–584	311	270–1,053	503	270–1,053
CD19 + /CD45 + (%)	**7**	9–18	**10**	13–26	13	13–26
CD19 + (cells/μL)	324	247–578	**398**	461–1,456	629	461–1,456
CD4 + /CD8 +	**0.413**	0.92–1.73	**0.47**	0.90–2.13	**0.8253**	0.90–2.13

Values higher or lower than the normal range were marked in bold. *The reference ranges suggested by previous publication (Ding et al., 2018).

### Genetic findings

In Family 1, Trio-WES was performed to detect a novel matrilineal heterozygous splice variant NM_138576.4: c.427 + 1G > A in the *BCL11B* gene of the proband (III-2), as further verified by performing Sanger sequencing (P8F/8R) in other family members. The same gene variant was observed in the proband’s brother (Family 1: III-4) rather than in her father (Family 1: II-4), younger sister (Family 1: III-3), her aunt (Family1: II-6), and maternal grandmother (Family 1: I-4) (Figure 1D). It indicates the occurrence of variant segregation in this family with the disorder. It was also shown in our study that the gene variant was non-existent in the ExAC, 1,000 Genomes Project and gnomAD. *In silico* prediction tools, Splice AI prediction was made that the c.427 + 1G > A variant could lead to the loss of the splice donor with a high score (0.99) and cause intron retention. In line with the ACMG guidelines, it was suggested that the gene variant should be classified as pathogenic (PVS1 + PP1 + PM2-Supporting). This variant has been submitted to the ClinVar database (Variation ID: 1292046). Besides, there were two other compound heterozygous variants of the *SLC26A4* gene detected as pathogenic gene variations in the proband’s father: NM_000441.2: c.1522A > G (p.Thr508Ala) and c.919-2A > G.

In Family 2, Trio-WES was carried out to detect a *de novo* heterozygosis variant NM_138576.2: c.2461_2462 ins GAGCCACACCGGCG(p.Glu821Glyfs*28)of the *BCL11B* gene in the proband (Family 2: II-1), as confirmed by Sanger sequencing (P9F/9R) (Figure 1E). In addition, we confirmed that the proband of Family 2 (II-1) and his parents (I-1 and I-2) were first-degree relatives. The results are provided in Supplementary Figure 1. The gene variation was identified in the last exon of the *BCL11B* gene and then predicted to escape non-sense-mediated mRNA decay (NMD), which probably resulted in a truncated protein, given the loss of the last two C-terminal DNA-binding zinc-finger domains. This gene variant did not exist in the ExAC, 1,000 Genomes Project, and gnomAD, nor was it reported in literature. According to the ACMG guidelines, this variant could be classified as pathogenic (PVS1_Strong + PS2 + PM2-Supporting). This variant has been submitted to the ClinVar database (Variation ID: 1292051).

### *In vitro* functional analysis

#### c.427 + 1G > A variant activates a cryptic splice site

**FIGURE 3:**
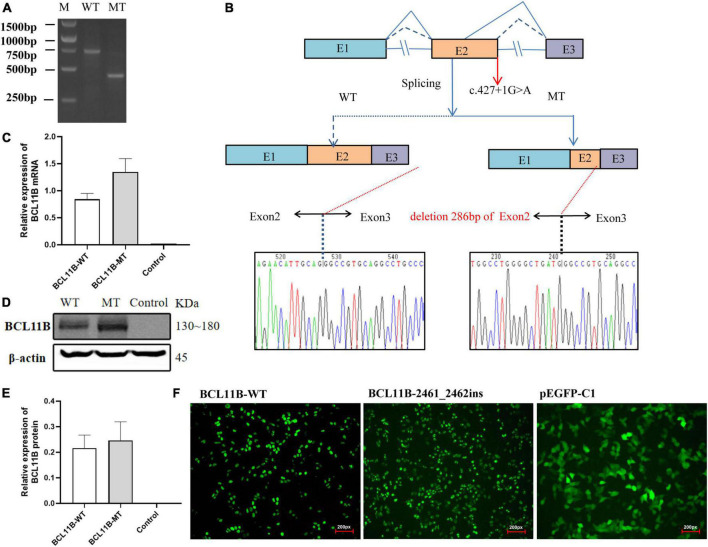
The *in vitro* minigene assay of the c.427 + 1G > A variant and the overexpression assay of the c.2461_2462insGAGCCACACCGGCG variant in HEK293T cells. **(A)** The identification of a shorter transcript with 286 bp deletion of the Exon 2 downstream, attributing to the activation of a novel cryptic 5′donor splice site in Exon 2. **(B)** A schematic diagram of the abnormal splice and Sanger sequencing of cDNA from transfected cells. **(C)** Quantitative analysis of mRNA expression of WT and frameshift mutant plasmids. **(D)** Western blot analysis of WT and frameshift mutant protein. **(E)** Quantitative analysis of protein expression of WT and frameshift mutant plasmids. **(F)** Fluorescent image (200px) of wild-type and mutant BCL11B-EGFP in HEK293T cells. Both wild-type and mutant types could be expressed, and there was no significant change in cell morphology and localization. M, DNA marker; WT, wild type; MT, mutant type.

For Family 1, an *in vitro* minigene splicing assay was performed to confirm whether the c.427 + 1G > A variant would affect normal splicing. The RT-PCR analysis of HEK-293T cells transfected with a mutant plasmid showed aberrant splicing in comparison with wild-type. As a result, the agarose gel electrophoresis of RT-PCR products displayed a single band (estimated 710 bp) from the wild type and a small band (estimated 420 bp) in the mutant type (Figure 3A). As revealed by the sequencing of the PCR fragment, the c.427 + 1G > A variant led to a shorter transcript with 286-bp deletion of downstream of Exon 2, which is attributed to the activation of a novel cryptic 5′donor splice site within Exon 2. Meanwhile, the 5′ donor splice site in Exon 3 was found normal, despite Exon 3 translation errors and premature termination. Represented as NM_138576.4: c.142_427del, the newly formed transcript was predicted to produce a prematurely truncated protein p.Val48Glyfs*14. A schematic diagram of the abnormal splice is shown in Figure 3B.

We repeated the minigene splicing assay in HepG2 cells and found that the abnormal shear results caused by the c.427 + 1G > A variant were consistent with those in HEK 293T cells. Gel electrophoresis and Sanger sequencing results are provided in the Supplementary Material (Supplementary Figures 2–4).

#### p.Glu821Glyfs*28 variant escape non-sense-mediated mRNA decay

As for Family 2, the mutant and wild-type recombinant plasmids were constructed and transfected into the HEK293T cells so as to better observe the effect of frameshift variant on protein expression. There was no significant difference revealed by QPCR in the level of mRNA between the mutant and the wild type (Figure 3C). Three independent replicates of WB showed that the protein carrying the p.Gl821GlyFs *28 variant could be expressed, and there was no significant difference between the mutant and the wild type (Figures 3D,E). Moreover, after transfection of wild-type and mutant types of BCL11B plasmids into 293T cells, EGFP fluorescence imaging showed that the nuclear localization and cell morphology of the mutant cells did not change significantly compared with the wild type (Figure 3F), which confirms the escape of the p.Glu821Glyfs*28 variant from NMD. This finding is the same as expected because this variant introducing the premature stop codon is located in the last exon of *BCL11B* gene escape NMD-scanning.

## Discussion

This study identified a new splice variant and a novel frameshift variant of the *BCL11B* gene in two unrelated pedigrees, including 3 patients in Family 1 and 1 patient in Family 2 with neurodevelopmental disorders. According to the *in vitro* minigene assay, the splice variant could cause the abnormal splicing of the *BCL11B* gene. Meanwhile, the extended phenotype with developmental retardation was observed in the proband of Family 1, suggesting the clinical heterogeneity and complex biological mechanisms of the disease.

It is widely known that *BCL11B*-related disorder can lead to two phenotypes with common features, namely, type 49 immunodeficiency and intellectual development disorders with malformation, speech delay, and T-cell abnormalities. There were 19 patients reported to show delayed psychomotor development, of whom only one showed the severe combined immune deficiency caused by missense variants (Punwani et al., 2016). In addition to the common features shown by all patients, such as facial dysmorphism, it was also found in this study that the proband in Family 1 also showed regression in intellectual and language development, which has been unreported previously. Moreover, the clinical manifestations of the 3 patients in Family 1 varied significantly. The proband showed more typical features of the disorder than her mother and brother did, whereas the younger brother exhibited milder symptoms. This finding suggested phenotypic heterogeneity of the splice site variant in *BCL11B*-related disorder from this family-based study. There are various reasons for this phenotypic heterogeneity. It is suspected that the detected splice variation may lead to various sets of abnormal transcripts in different patients, which is associated with the significant variation in gene expression and function. Additionally, the other variants might be excluded from WES analysis, which is suspected to be a pathogenic factor located in non-coding regions or associated with complex structural rearrangements. For the proband’s younger brother, the clinical phenotype showed changes due to aging. A follow-up survey or gene investigation could be conducted to clarify this.

At present, the diverse roles of *BCL11B* in T cell biology and Group 2 innate lymphoid cell (ILC2) development have been studied by many researchers (Walker et al., 2015; Yu et al., 2015, 2016; Xu et al., 2019). It was in 2016 that the first patient manifesting severe combined immunodeficiency (SCID) and multisystem anomalies was reported (Punwani et al., 2016), with nearly half of the reported patients displaying persistent infections, eosinophilia, allergy, and asthma. Consistently, it was discovered in our study that the proband and her younger brother in Family 1 exhibited persistent infections as well, and that the proband in Family 2 showed only allergy. Moreover, flow cytometric analysis was conducted to reveal that the ratio of CD4 + /CD8 + was the lowest in the proband and her younger brother of Family 1, but normal in the proband in Family 2. According to the study of Lessel et al. (2018) this is linked to the impairment of T cell immunity. Given a lack of definitive immune deficiency diagnosis, it is necessary to reveal the impact of *BCL11B* gene variation on the immune system through more clinical cases and pathogenic mechanism studies.

*De novo* cryptic splice variants contribute significantly to rare genetic disorders, especially intellectual disability and autism spectrum disorders (2015; Jaganathan et al., 2019). In this study, a minigene expression assay was performed to figure out the *in vitro* effects of the c.427 + 1G > A variant on gene function of *BLC11B*. By causing the absence of the splice donor on Intron 2 and the incidence of a novel cryptic donor splice site on Exon 2, the splice variant shortened Exon 2 and led to the generation of an abnormal pre-mRNA (c.142_427del). On the translation level, the abnormal pre-mRNA produced a prematurely truncated protein p.Val48Glyfs*14 but with no ZnF_C2H2 domains of the BCL11B protein. Given the involvement of ZnF2 and ZnF3 domains in DNA binding (Kominami, 2012), it is speculated that the c.427 + 1G > A gene variant could interfere with the recognition of BLC11B protein and DNA and the interaction between them. Besides, the abnormal truncated variant p.Val48Glyfs*14 is located in Exon 2, and it is thus predicted to trigger NMD and cause haploinsufficiency as a result. In summary, the genetic etiology of patients in Family 1 is closely associated with the splice variant c.427 + 1G > A of the *BCL11B* gene.

It is also known that *de novo* frameshift variants can result in severe intellectual disability and developmental delay (Kosmicki et al., 2017). For all the *BCL11B* variants associated with developmental delay and intellectual disability, frameshift variants account for 76% (13/17), with nearly all of them being *de novo.* However, there was only one frameshift variant p.Asp534Thrfs*29 reported as hereditary (Lessel et al., 2018). In our study, the proband in Family 2 carried a *de novo* frameshift variant p.Glu821Glyfs*28 in the *BCL11B* gene. As confirmed by the overexpression experiments, this variant could escape NMD to form a truncated protein, despite no significant difference observed in protein and RNA expression between the mutant plasmid and the wild-type plasmid. So far, the variant p. (Gly820Alafs*27) has been confirmed to result in a functional null allele and suppress progenitor cell proliferation in hippocampal of Bcl11b mutant mice. Also, it has been found to interfere with the expression of the BCL11B in dentate neurons (Lessel et al., 2018). As a neighboring variant, the p.Glu821Glyfs*28 truncated variant as identified in this study has a potential to produce a functional null allele, thus causing the disorder. By summarizing the phenotypes of all patients with a frameshift/non-sense variant (Supplementary Table 3), it can be found out that all the frameshift variants may cause the loss of the last two or three C-terminal ZnF_C2H2 domains, except for p.Ala891Profs *106 and p.Cys81Leufs*76 variants. As a result, NMD can be activated (Hamby et al., 2011). However, there was no significant correlation observed between the number of retained Zn finger domains and the severity of symptoms. Since the zinc finger domain, usually, must be connected in series for binding DNA (Avram et al., 2002), the loss of the zinc finger domain may offset the physiological impact of BCL11B protein by affecting its transcription-regulatory activity.

## Conclusion

In conclusion, a novel splicing variant of the *BCL11B* gene and a novel frameshift variant were identified in this study from two different families separately. The findings of this study expand the spectrum of gene variation associated with the disease. The genetic and clinical data used in the study provide evidence for a study to better understand the pathogenic mechanism and genetic etiology of the disease. *BCL11B*-related disorder is an intellectual development disorders characterized by malformation, speech delay, and T-cell abnormalities or immune deficiency. Genetic testing is conducive to improving the accuracy of a clinical diagnosis and providing firm evidence for gene counseling.

## Data availability statement

The original contributions presented in this study are included in the article/Supplementary material, further inquiries can be directed to the corresponding author/s.

## Ethics statement

The studies involving human participants were reviewed and approved by the Ethics Committee of the Xi’an Children’s Hospital. Written informed consent to participate in this study was provided by the participants; legal guardian/next of kin. Written informed consent was obtained from the individual(s), and minor(s); legal guardian/next of kin, for the publication of any potentially identifiable images or data included in this article.

## Author contributions

FC and YY contributed to conception and design of the study. XT and LZ collected the clinical data. FC and XT wrote the first draft of the manuscript. HL revised the manuscript, while XZ and MD coordinated and supervised data collection. All authors contributed to revising the manuscript and read through and approved the submitted version.
