# Supplementary material for: Identification of two novel variants of the BCL11B gene in two Chinese pedigrees associated with neurodevelopmental disorders
Source: Front Mol Neurosci. 2022 Sep 13;15:927357. doi: 10.3389/fnmol.2022.927357 (PMC9513357; doi:10.3389/fnmol.2022.927357)
Supplement: Supplementary file 1 [file Data_Sheet_1.doc]

| Primers | Sequencing |
| --- | --- |
| P1F | AAGCTTGGTACCGAGCTCGGATCCATGTCCCGCCGCAAACAGGGCAACCCGC |
| P1R | CCCAGGTGAAGCGCGGACCCACGCGCTCGCCAACT |
| P2F | TGGGTCCGCGCTTCACCTGGGGAGTCTGAAAGACT |
| P2R | ACCTTGGGTCATGCCCATCTTGCTCCAGCACAGGC |
| P3F | AAGATGGGCATGACCCAAGGTGTCCCGGGCCTGTA |
| P3R | TTAAACGGGCCCTCTAGACTCGAGCTGACAACTGACACTGGCATCCAAAGGG |
| P4F | GATATACACTGTTTGAGATGAGGA |
| P4R | ACCTGGATGGTGGACCCTCA |
| P5F | GGCTAACTAGAGAACCCACTGCTTA |
| P5R | CTGACAACTGACACTGGCATCCA |
| P6F | 5’-CGgagccacaccggcgAGCGGCCTTACAAGTGCGA-3’ |
| P6R | 5’-CTcgccggtgtggctcCGCCGGTGTGGCTCCGCC-3’ |
| P7F | 5’-GCAGCTTGGGTGCCTGCTA-3’ |
| P7R | 5’-GATGCCTTTCGTGGGTGAGA-3’. |
| human-b-actin-F | 5’-CCTGGCACCCAGCACAAT-3’; |
| human-b-actin-R | 5’-GGGCCGGACTCGTCATAC-3’ |
| P8F | ACAAGGACAGCCCGCCAC |
| P8R | CCTCACCAAAAGCAAGCAGC |
| P9F | TTCCTGGGCTTCACGGACGC |
| P9R | CGCAGACACAGGTTAGGTTGG |

**Supplemental Tables**

**Table 1**  List of all primers for this study

**TABLE 2**  Humoral immunoassay of the patients in this study

| immunoglobulin | Family 1: III-2 (Proband) | Family 1: III-4(young brother) | Family 2: Proband | Normal Value |
| --- | --- | --- | --- | --- |
| IgG | 16.7 | 11.6 | 13.00 | 3.41-19.6(g/L) |
| IgM | **2.64** | 0.86 | 1.29 | 0.43-1.63(g/L) |
| IgA | **4.5** | 1.46 | 1.89 | 0.56-3.43(g/L) |

**TABLE 3** The clinical features of frameshift variants of the *BCL11B* gene in published patients and the proband of family 2 in this study

| Variants | Eoxn | zinc finger  domain | Clinical features | | | | | | | | | | | Refs. (first reported) |
| --- | --- | --- | --- | --- | --- | --- | --- | --- | --- | --- | --- | --- | --- | --- |
|  |  |  | Intellectual  disability | Speech  impairment | Delay motor  development | Autistic  features | Facial  abnormalities | Dental  anomalies | Refractive  error | Feeding  diffificulties | Abnormal  MRI | Immune  response | Allergy/  asthma |  |
| c.242delG (p.C81Lfs*76) | 2 | / | + | + | + | - | + | + | Myopia | - | - | Frequent/  atypica | - | Lessel, et al |
| c.1192_1196delAGCCC (p.S398Qfs*117) | 4 | ZnF1 | + | + | + | - | + | - | - | - | + | Deficiency  of naïve T-cells | - | Yang,et al |
| c.1365_1367delCAA (p.Y455*) | 4 | ZnF1+ZnF2 | + | + | + | - | + | - | Exotropia | - | - | - | + | Lessel, et al |
| c.1495G>T (p.E499*) | 4 | ZnF1+ZnF2+ZnF3 | + | + | + | - | + | - | Myopia | + | + | - | + | Lessel, et al |
| c.1502dupG (p.T502Hfs*15) | 4 | ZnF1+ZnF2+ZnF3 | + | + | + | - | + | - | - | - | - | - | - | Lessel, et al |
| c.1552delC (p.R518Afs*45) | 4 | ZnF1+ZnF2+ZnF3 | + | + | + | - | + | + | - | - | - | Frequent  infections | + | Lessel,et al |
| c.1600delG (p.D534Tfs*29) | 4 | ZnF1+ZnF2+ZnF3 | + | + | + | + | + | - | - | - | - | - | - | Lessel ,et al |
| c.1887_c.1893delCGGCGGGC  （p. G629Gfs*92） | 4 | ZnF1+ZnF2+ZnF3 | + | + | + | - | + | + | Esotropia | - | + | Frequent  infections | - | Yan et al. |
| c.1944_1965d  elGGCGCG  GTCAACGG  GCGCGGGG (p.G649Afs*67) | 4 | ZnF1+ZnF2+ZnF3 | + | + | + | - | + | + | - | - | - | Frequent  infections | - | Lessel, et al |
| c.2190_2200delGGACGCACGAC(p.T730Tfs*151) | 4 | ZnF1+ZnF2+ZnF3 | + | + | + | - | + | - | - | - | - | Low TREC at  birth | - | Qiao, et aL |
| c.2449_2456 dupAGCC  ACAC(p.G820Afs*27) | 4 | ZnF1+ZnF2+ZnF3+ZnF4 | + | + | + | + | + | + | Hyperopia | - | - | - | - | Lessel, et al |
| c.2461_2462insGAGCCACACCGGCG（p.E821Gfs*28） | 4 | ZnF1+ZnF2+ZnF3+ZnF4 | + | + | + | - | + | + | - | + | - | - | + | present |
| c.2671delG  (p.A891Pfs*106) | 4 | ZnF1+ZnF2+ZnF3+Zn4+ZnF5+ZnF6 | + | + | + | + | + | - | Hyperopia | + | - | Frequent  infections | + | Lessel, et al |
