# Supplementary material for: Identification of two novel variants of the BCL11B gene in two Chinese pedigrees associated with neurodevelopmental disorders
Source: Front Mol Neurosci. 2022 Sep 13;15:927357. doi: 10.3389/fnmol.2022.927357 (PMC9513357; doi:10.3389/fnmol.2022.927357)
Supplement: Supplementary file 2 [file Data_Sheet_2.doc]

1. **The phylogenetic analysis of family 2**

According to the SNP results from Trio-WES sequencing of family 2 samples, PLINK tool was used to convert the VCF files into binary files, and the --related parameter of King software was used to obtain the family relationship results, and R language was used to visualize the results. The results showed that the proband of family 2 (II-1) and his parents (I-1 and I-2) were first-degree relatives, and the values were 0.2641 and 0.2677, respectively (the threshold of first-degree relatives ranged from 0.177 to 0.354). The results are provided in the Figure S1.


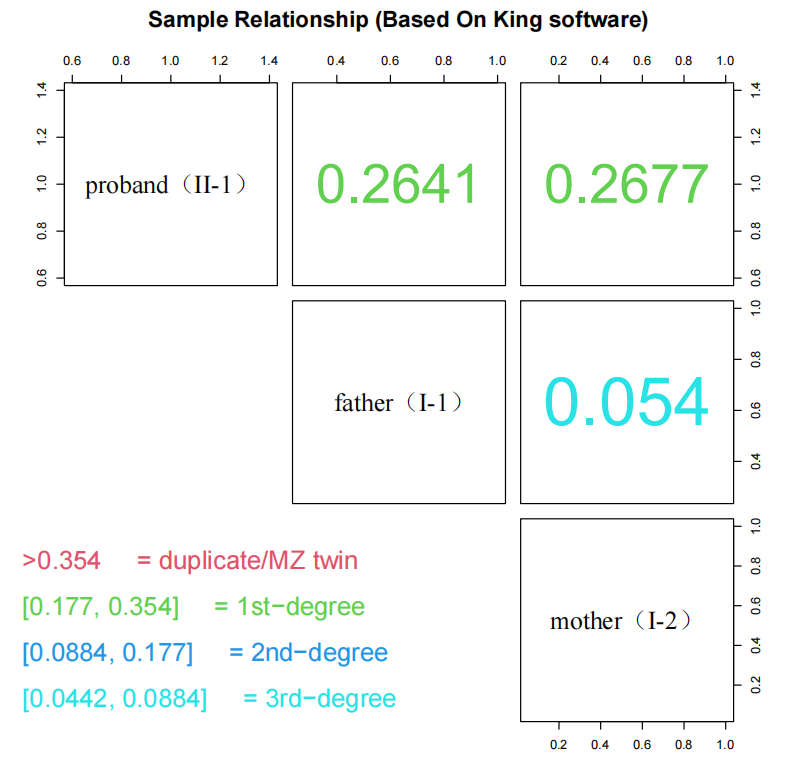


Figure S1 Diagram of the results of phylogenetic analysis in family 2 by King software

**2.Minigene splicing assay of** **c.427+1G>A variant of *BCL11B***

The recombinant plasmids pMini-CopGFP-BCL11B-wt (wild-type) and pMini-CopGFP-BCL11B-mt (c.427+1G>A) were transfected into HepG2 cells. With total RNA extracted and reverse-transcribed into cDNA after 48 hours of transfection, reverse transcription polymerase chain reaction (RT-PCR) was triggered by a primer pair of P5F/5R. The results of Gel electrophoresis and Sanger sequencing of RT-PCR products are showed in Figure S2, S3 and S4.


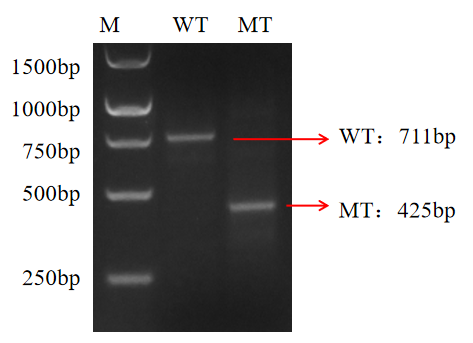


Figure S2 The Gel electrophoresis of RT-PCR products displayed a single band (estimated 710bp) from the wild type (WT) and a small band (estimated 420 bp) in the mutant type(MT) .


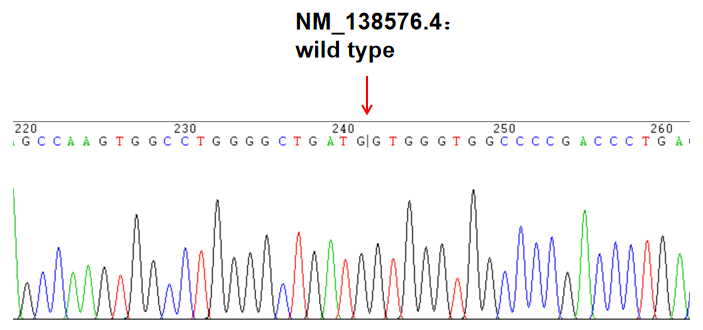


Figure S3 The cDNA Sanger sequencing of *BCL11B* gene (wide type)


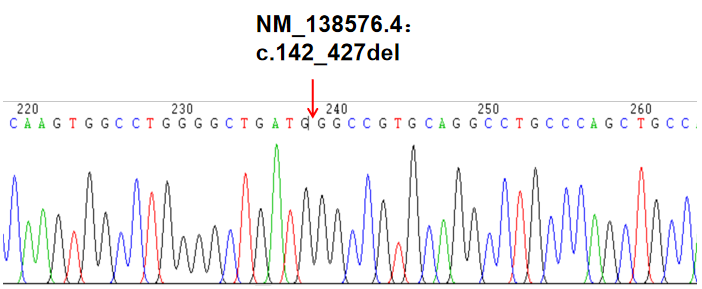


Figure S4 The cDNA Sanger sequencing of c.427+1G>A variant of *BCL11B* gene (wide type). The c.427+1G>A variant led to a shorter transcript with 286bp deletion of downstream of exon 2, which is attributed to the activation of a novel cryptic 5’donor splice site within exon 2.
